# Supplementary material for: Oxidative Stress Markers in Inflammatory Bowel Diseases: Systematic Review
Source: Diagnostics (Basel). 2020 Aug 17;10(8):601. doi: 10.3390/diagnostics10080601 (PMC7459713; doi:10.3390/diagnostics10080601)
Supplement: Supplementary file 1 [file diagnostics-10-00601-s001.pdf]

Supplementary materials

Table S1. Prooxidants/oxidative stressors as potential biomarkers in inflammatory bowel diseases

Analyte	Type	Disease	Evaluated population[1]	Findings[2]	Potential	Ref.
Arg 1 (mRNA)	T-E	CD, UC	FFPE slides: 25 CD (10); 38 UC (21); 25 HC	↓ in aCD vs. HC; ↑ in aUC vs. iUC and HC; ↑ in aUC vs. aCD	aCD marker aUC marker $Diff_{aCD/aUC}$	[29]
Cu	BS	IBD	19 UC (aa/RI, ns); 16 CD (aa/ CDAI≥150); 30 HC	↑ in IBD in females	aIBD marker in females	[25]
COX2 (mRNA)	T-E	CD, UC	FFPE slides: 25 CD (10); 38 UC (21); 25 HC	↑ in aCD vs. iCD and HC; ↑ in aUC vs. iUC and HC	aIBD marker IBD activity	[29]
NOS2 (mRNA)	T-E	CD, UC	FFPE slides: 25 CD (10); 38 UC (21); 25 HC	↑ in aCD vs. iCD and HC ↑ in aUC vs. iUC and HC	aIBD marker IBD activity	[29]
NOX2 (mRNA)	T-E	CD, UC	FFPE slides: 25 CD (10); 38 UC (21); 25 HC	↑ in aCD vs. iCD and HC ↑ in aUC vs. iUC and HC	aIBD marker IBD activity	[29]
PP (StP)	BP	CD	52 CD (37/CDAI≥150); 99 HC	↓ in aCD vs. iCD and HC; CDAI -0.54; CRP -0.54; IL-6 -0.57; Chol 0.70; TG 0.52	CD activity	[45]
NO	saliva	CD	28 CD (ns/CDAI, ns); 20 HC	↑ in CD	CD marker	[17]
IoFRP	T-H	CD	45 CD (aa/ns); 30 IBS; IS	↑ in CD vs. IBS	$Diff_{CD/IBS}$	[110]
RoPRG	T-H	CD	45 CD (aa/ns); 30 IBS; IS	↑ in CD vs. IBS	$Diff_{CD/IBS}$	[110]
StP	T-H	CD	45 CD (aa/ns); 30 IBS; IS	↑ in CD vs. IBS	$Diff_{CD/IBS}$	[110]
DUOX2 (E.C. 1.6.3.1)	T-M	CD, UC	25 pediatric CD (aa/PCDAI, ns); 22 pediatric UC (aa/PUCAI, ns); 24 GIS	↑ in UC in AsC (22×), DeC (12.5×), and TI (13.5×)[3]	$Diff_{CD/UC}$	[106]
LOX15 (E.C. 1.13.11.33)	T-M	CD, UC	25 pediatric CD (aa/PCDAI, ns); 22 pediatric UC (aa/PUCAI, ns); 24 GIS	↑ in UC in AsC (25×), DeC (13×), and TI (18×)[3]	$Diff_{CD/UC}$	[106]
LOX5 (E.C. 1.13.11.34)	T-M	CD, UC	25 pediatric CD (aa/PCDAI, ns); 22 pediatric UC (aa/PUCAI, ns); 24 GIS	↑ in IBD vs. GIS; (8.2× in AsC, 9× in DesC, 11.6× in TI)[3]	$Diff_{IBD/GIS}$	[106]
NOS2 (E.C. 1.14.13.39)	T-M	CD, UC	25 pediatric CD (aa/PCDAI, ns); 22 pediatric UC (aa/PUCAI, ns); 24 GIS	↑ in IBD vs. GIS (16.3× in AsC, 9.5× in DesC, 5.3× in TI)[3] ↑ in UC in AsC (3.5×), DeC (3×), and TI (2.7×)[3]	$Diff_{IBD/GIS}$ $Diff_{CD/UC}$	[106]
Cu	BW	IBD	167 IBD (132; CDAI≥150 or CAI>5; 100 CD and 67 UC); 45 HC	↑ in aIBD vs. HC ↑ in aIBD vs. iIBD	IBD marker IBD activity	[24]
NO	saliva	CD, UC	16 CD (ns); 16 UC (ns); 16 HC	↑ in CD vs. HC; ↑ in UC vs. HC	IBD marker	[16]
NO	T-H	CD, UC	22 UC (15/ ns), paired biopsies inf. & non-infl. colon (n=6 aUC); 11 CD (6/ns); 14 specific colitis (infl.); 10 GIS	↑ in aUC and iUC vs. GIS; ↑ in aCD and iCD vs. GIS; ↑ in spec. colitis vs. GIS; ↑ in aUC/aCD vs. iUC/iCD ↑ in infl. and non-infl. aUC vs. iUC and GIS; If stratified by severity into: GIS/iIBD/miIBD/moIBD/sIBD, r=0.81	IBD marker IBD activity $Dif_{IBD/GIS}$	[19]
TOC	BS	CD, UC	40 CD (ns/CDAI, ns); 40 UC (ns/RI-EAI, ns); 80 HC	↑ in CD and UC vs. HC; CDAI 0.96; RI-EAI 0.93	IBD marker MI	[20]
NOS2	T-I	UC	16 aUC; 14 UC+N (9 D, 14 UCAC); 17 NM	↑ in aUC and UC+N vs. NM	Inflam./N	[30]

(E.C. 1.14.13.39)						
Cu	BP	CD, UC	20 CD (ns); 20 UC (ns); 50 HC	n/a; CRP[4] 0.86	none	[26]
OSI	BP	IBD	71 pediatric IBD (47/PCDAI or PUCAI; 35 CD and 36 UC); 29 GIS	No association	none	[22]
TOC	BP	IBD	71 pediatric IBD (47/PCDAI or PUCAI; 35 CD and 36 UC); 29 GIS	No association	none	[22]
MPO (E.C. 1.11.2.2.)	BS	UC	30 UC (aa/TW, ns); 30 HC	↑ in UC n/a with severity, CRP, ESR	UC marker	[27]
NO	saliva	UC	37 UC (aa/TW, ns); 15 HC	↑ in UC; age 0.38; n/a with UC activity, extent, treatment	UC marker	[18]
OSI	BP	UC	20 UC (11/RI, ns); 20 HC	↑ in UC; n/a with UC activity; CRP 0.41; ESR 0.54	UC marker	[23]
TOC	BP	UC	20 UC (11/RI, ns); 20 HC	↑ in UC; n/a with UC activity; CRP 0.42; ESR 0.42	UC marker	[23]
SMO (E.C. 1.5.3.16)	T-H	UC	mRNA: 51 UC & 14 NM; IHC: 53 UC & 16 NM; MDAI	↑ in UC (mRNA); ↑ in UC (immune cells; IHC); ↑ immunoreactivity with ↑ severity: MDAI 0.65, EA, HA	UC marker UC activity MI	[28]

[1], number of patients (number of patients with active disease/scoring system≥cut-off for active disease); [2], data presented as ↑ increased or ↓decreased levels between indicated groups and as correlation coefficients preceded by the variable; [3], data showing fold change in expression and analyzed separately for three bowel fragments: AsC=ascending colon, DeS=descending colon, TI=terminal ileum; [4] correlation in CD patients; ns, not specified; aa, all active; n/a, no association; inflam., inflamed; NO, nitric oxide; MPO, myeloperoxidase; SMO, spermine oxidase; COX2, inducible cyclooxygenase; NOX2, NADPH oxidase; NOS2, inducible NO synthase; LOX, lipoxygenase; DUOX2, dual oxidase 2; Arg 1, arginase 1; TOC, total oxidant capacity; OSI, oxidative stress index calculated as total oxidant capacity/total antioxidant status; IoFRP, intensity of free radical processes determined as sum of spontaneous chemiluminescence; RoPRG, rate of peroxide radical generation; PP, peroxidation potential; StP, susceptibility to undergo peroxidation; Cu, copper; HS, healthy controls; CD, Crohn's disease; UC, ulcerative colitis; IBD, inflammatory bowel diseases; iIBD, inactive IBD; miIBD, mild IBD; moIBD, moderate IBD; sIBD, severe IBD; aCD, active CD; iCD, inactive CD; aUC, active UC; iUC, inactive UC; N, neoplasms; D, dysplasia; NM, normal mucosa; UCAC, UC-associated cancer, GIS, non-IBD patients with gastrointestinal symptoms; IBS, irritable bowel syndrome; MI, mucosal inflammation; $Diff_{CD/UC}$; differential marker for CD and UC; $Diff_{IBD/GIS}$; differential marker for IBD and GIS; $Diff_{CD/IBS}$; differential marker for CD and IBS; $Diff_{aCD/aUC}$; differential marker for aCD and aUC; CDAI, Crohn's disease activity index, CAI, colitis activity index; MDAI, Mayo disease activity index; PCDAI, pediatric Crohn's disease activity index; PUCAI, pediatric ulcerative colitis index; RI-EAI, Rachmilewitz endoscopic activity index; RI, Rachmilewitz index; TW, Truelove-Witt index; HA, histopathological activity; ES, endoscopic activity; FFPE, formalin-fixed paraffin-embedded; ESR, erythrocyte sedimentation rate; Chol, total cholesterol; TG, triglycerides; IS, interventional study; T-I, tissue-based marker determined with immunohistochemistry (IHC); T-E, tissue-based marker analyzed as mRNA expression; T-M, tissue metaproteomics – aspirates collected from mucosal-luminal interface for the analysis of microbial and human proteins; BS, blood-based marker determined in serum; BE, blood-based marker determined in erythrocytes; BP, blood-based marker determined in plasma; BW, blood-based marker determined in whole blood; T-H, tissue-based marker determined in homogenates.

Table S2. Enzymatic antioxidants as potential biomarkers in inflammatory bowel diseases

Analyte	Type	Disease	Evaluated population[1]	Findings[2]	Potential	Ref.
$PON1_P$ (E.C: 3.1.8.1)	BS	UC	30 UC (aa/TW, ns); 30 HC	↓ in UC; n/a with activity	UC marker	[48]
$PON1_P$ (E.C: 3.1.8.1)	BS	UC	66 UC (MDAI: mild≤5advanced); 24 HC	n/a; CRP as indep. pred. of $PON1_P$	none	[46]
$PON1_A$ (E.C. 3.1.1.2)	BS	UC	66 UC (MDAI: mild≤5advanced); 24 HC	↓ in UC; MDAI and WBC indep. pred. of $PON1_A$	UC marker	[46]
GPx (E.C. 1.11.1.9)	BP	CD	37 CD (26/CDAI≥150); 37 HC	No associations	none	[43]
GPx (E.C. 1.11.1.9)	BS	IBD	14 CD (8/CDAI≥150); 27 UC (13/MDAI≥4); 18 HC	↓ in iIBD vs. aIBD and HC	IBD activity	[38]
GPx (E.C. 1.11.1.9)	BP	CD	47 CD (25/CDAI≥150); 25 HC	No association	none	[42]

GPx (E.C. 1.11.1.9)	saliva	CD	47 CD (25/CDAI≥150); 25 HC	No association	none	[42]
GPx (E.C. 1.11.1.9)	BE	IBD	91 IBD (43/CDAI & TW); 45 HC	No associations	none	[34]
GPx (E.C. 1.11.1.9)	BE	CD	15 pediatric CD (ai/PCDAI, ≥10); 15 HC; IS	n/a; PCDAI: -0.46	CD activity	[33]
GPx (E.C. 1.11.1.9)	BS	IBD	30 IBD (CDAI & LI); 30 HC	↑ in IBD; CAL 0.55	IBD marker	[40]
GPx (E.C. 1.11.1.9)	BP	CD, UC	35 UC (13/RI, ns); 12 CD (6/HBI, ns); 30 HC	↑ in aCD and iCD vs. HC; ↑ in aUC and iUC vs. HC	IBD marker	[39]
GPx (E.C. 1.11.1.9)	BP	CD	43 CD (16/CDAI, >150); 15 HC	↑ in aCD vs. HC	aCD marker	[41]
GPx (E.C. 1.11.1.9)	BL	CD	25 adult CD (ns/CDAI≥150); 88 HC; 21 pediatric CD (ns/PCDAI, ns); 11 HC	n/a; PCDAI –0.51	CD activity	[35]
GPx (E.C. 1.11.1.9)	T-E	IBD	12 CD (ns); 12 UC (ns)	↑ in infl. vs. non-infl.	MI	[118]
GPx (E.C. 1.11.1.9)	T-H	CD	20 CD (18 infl. and 14 non-infl.); 16 NM	↓ in infl. vs. non-infl. and NM	MI	[83]
mGPx (E.C .1.11.1.9)	T-M	CD, UC	25 pediatric CD (aa/PCDAI, ns); 22 pediatric UC (aa/PUCAI, ns); 24 GIS	↑ in IBD vs. GIS; 4× (AsC), 14× (DeC), 11× (TI)[3]	$Diff_{IBD/GIS}$	[106]
CAT (E.C. 1.11.1.6)	BE	CD	15 pediatric CD (ai/PCDAI, ≥10); 15 HC; IS	No associations	none	[33]
CAT (E.C. 1.11.1.6)	BE	UC	81 UC (ns/AI≥150); 85 HC	↑ in UC vs. HC; n/a with activity	UC marker	[32]
CAT (E.C. 1.11.1.6)	T-H	CD, UC	12 CD (ns/ HBI≥8) 5 UC (ns/ MDAI≥6); 12 HC	No associations	none	[44]
CAT (E.C. 1.11.1.6)	BS	IBD	30 IBD (CDAI & LI); 30 HC	↑ in IBD; CAL 0.52	IBD marker	[40]
CAT (E.C. 1.11.1.6)	BL	CD	25 aCD (HBI≥5)→19 rCD[4]; 20 iCD; 25 HC	↓ in aCD, iCD, and rCD vs. HC	CD marker	[36]
CAT (E.C. 1.11.1.6)	BL	CD	58 CD (32/CDAI≥150); 26 HC	↓ in aCD vs. iCD and HC	CD activity	[37]
CAT (E.C. 1.11.1.6)	BP	CD, UC	20 CD (ns); 20 UC (ns); 50 HC	No associations	none	[26]
SOD (E.C. 1.15.1.1)	BL	CD	25 aCD (HBI≥5)→19 rCD; 20 iCD; 25 HC	↑ in aCD vs. iCD, rCD, and HC	aCD marker CD activity	[36]
SOD (E.C. 1.15.1.1)	BE	CD, UC	93 UC (42/RI, ns) 81 CD (53/ CDAI≥150); 105 HC	n/a; CDAI -0.29; ESR[5] -0.25	none	[31]
SOD (E.C. 1.15.1.1)	BE	CD	15 pediatric CD (ai/PCDAI, ≥10); 15 HC; IS	↓ in CD; CAL -0.37	CD marker	[33]
SOD (E.C. 1.15.1.1)	BE	UC	81 UC (ns/AI≥150); 85 HC	↑ in UC vs. HC; n/a with activity	UC marker	[32]
SOD (E.C. 1.15.1.1)	BE	CD	25 adult CD (ns/CDAI≥150); 88 HC; 21 pediatric CD (ns/PCDAI, ns); 11 HC	↑ in adult CD vs. HC	CD marker	[35]
SOD (E.C. 1.15.1.1)	BS	IBD	14 CD (8/ CDAI≥150) 27 UC (13/ MDAI≥4); 18 HC	↓ in iIBD vs. aIBD and HC	IBD activity	[38]
SOD (E.C. 1.15.1.1)	BS	IBD	19 UC (aa/RI, ns); 16 CD (aa/CDAI, ≥150); 30 HC	↓ in IBD	aIBD marker	[25]
SOD (E.C. 1.15.1.1)	BP	CD	47 CD (25/CDAI≥150); 25 HC	↓ in aCD vs. iCD and HC; CDAI -0.46; CRP -0.48	CD activity	[42]
SOD (E.C. 1.15.1.1)	saliva	CD	47 CD (25/CDAI≥150); 25 HC	n/a; CRP 0.37	none	[42]
SOD (E.C. 1.15.1.1)	BP	CD, UC	20 CD (ns); 20 UC (ns); 50 HC	n/a; CRP[5] 0.70	none	[26]
SOD[6] (E.C. 1.15.1.1)	T-H	UC	20 UC (paired infl. / non-infl.); 4 HC (NM)	↑ in infl. vs. non-infl.	MI	[119]

SOD (E.C. 1.15.1.1)	T-M	CD, UC	25 pediatric CD (aa/PCDAI, ns); 22 pediatric UC (aa/PUCAI, ns); 24 GIS	↑in CD in DeC (2.1×) and in TI (4.8×), no difference in AsC[3]	$Diff_{CD/UC}$	[106]
Prdx4[6] (E.C. 1.11.1.24)	T-H	UC	20 UC (paired infl. / non-infl.); 4 HC (NM)	↑ in infl. vs. non-infl.	MI	[119]
Prdx6[6] (E.C. 1.11.1.24)	T-H	UC	20 UC (paired infl. / non-infl.); 4 HC (NM)	↓ in infl. vs. non-infl.	MI	[119]
Prdx2 (E.C. 1.11.1.24)	T-M	CD, UC	25 pediatric CD (aa/PCDAI, ns); 22 pediatric UC (aa/PUCAI, ns); 24 GIS	↑in UC in AsC (7.2×), DeC (1.8×) and TI (1.9×)[3]	$Diff_{CD/UC}$	[106]
Prdx3[6] (E.C. 1.11.1.24)	T-H	UC	20 UC (paired infl. / non-infl.); 4 HC (NM)	↓ in infl. vs. non-infl.	MI	[119]
Prdx3 (E.C. 1.11.1.24)	T-M	CD, UC	25 pediatric CD (aa/PCDAI, ns); 22 pediatric UC (aa/PUCAI, ns); 24 GIS	↑in CD in DeC (1.5×) and in TI (2.7×) and ↑in UC in AsC (1.4×)[3]	$Diff_{CD/UC}$	[106]
FOCP & SEACP (E.C. 1.16.3.1)	BS	CD	14 CD patients (aa/ CDAI, ns); 52 HC; IS	↓ in aCD vs. HC	aCD marker	[55]
CP_P	BP	CD, UC	20 CD (ns); 20 UC (ns); 50 HC	No association	none	[26]
CP_P	T-M	CD, UC	25 pediatric CD (aa/PCDAI, ns); 22 pediatric UC (aa/PUCAI, ns); 24 GIS	↑ in IBD vs. GIS; (46× in AsC, 29× in DeC, 37× in TI)[3]; ↑ in CD in DeC (1.4×) and TI (5.7×) but ↑ in UC in AsC (6.2×)[3]	$Diff_{IBD/GIS}$ $Diff_{CC/UC}$	[106]

[1], number of patients (number of patients with active disease/scoring system≥cut-off for active disease); [2], data presented as ↑ increased or ↓decreased levels between indicated groups and as correlation coefficients preceded by the variable; [3], data showing fold change in expression and analyzed separately for three bowel fragments: AsC=ascending colon, DeS=descending colon, TI=terminal ileum; [4], patients with active disease were followed until they have achieved remission; [5] correlation in CD patients; [6], determined as protein using 2-dimensional gel electrophoresis (2-DGE)-based proteomics coupled with mass spectrometry; ns, not specified; aa, all active; ai, all inactive; n/a, no association; inflam., inflamed; indep. pred., independent predictor; PON1, paraoxonase-1 (A in superscript indicates the arylesterase activity and P – paraoxonase activity); GPx, glutathione peroxidase; mGPx, microbial GPx; CAT, catalase; SOD, superoxide dismutase; Prdx, thioredoxin-dependent peroxide reductase; FOCP, ferroxidase activity of ceruloplasmin; SEACP, specific activity of FOCP (ratio between ferroxidase activity and apoceruloplasmin); CP_P, ceruloplasmin determined as protein; HS, healthy controls; CD, Crohn's disease; UC, ulcerative colitis; IBD, inflammatory bowel diseases; aIBD, active IBD; iIBD, inactive IBD; miIBD, mild IBD; moIBD, moderate IBD; sIBD, severe IBD; aCD, active CD; iCD, inactive CD; rCD, remission CD; aUC, active UC; iUC, inactive UC; N, neoplasms; D, dysplasia; NM, normal mucosa; UCAC, UC-associated cancer; GIS, non-IBD patients with gastrointestinal symptoms; MI, mucosal inflammation; $Diff_{CD/UC}$; differential marker for CD and UC; $Diff_{IBD/GIS}$; differential marker for IBD and GIS; CDAI, Crohn's disease activity index, MDAI, Mayo disease activity index; PCDAI, pediatric Crohn's disease activity index; PUCAI, pediatric ulcerative colitis index; RI, Rachmilewitz index; LI, Lichtiger index; HBI, Harvey-Bradshaw index; AI, activity index defined by authors in their paper; TW, Truelove-Witt index; CRP, C-reactive protein; ESR, erythrocyte sedimentation rate; CAL, fecal calprotectin; IS, interventional study; T-E, tissue-based marker analyzed as mRNA expression; T-M, tissue metaproteomics – aspirates collected from mucosal-luminal interface for the analysis of microbial and human proteins; BS, blood-based marker determined in serum; BE, blood –based marker determined in erythrocytes; BP, blood-based marker determined in plasma; BL, blood-based marker determined in leukocytes; T-H, tissue-based marker determined in homogenates

Table S3. Non-enzymatic protein antioxidants as potential markers in inflammatory bowel diseases

Analyte	Type	Disease	Evaluated population[1]	Findings[2]	Potential	Ref.
F-SH	saliva	CD	28 CD (ns/CDAI, ns); 20 HC	Insignificantly lower in CD	none	[17]
F-SH	BS	UC	30 UC (aa/TW, ns); 30 HC	↑ in UC; n/a with activity, CRP or ESR	UC marker	[27]

F-SH	BP	IBD	91 IBD (43/CDAI & TW); 45 HC	↓ in aIBD vs. iIBD and HC	aIBD marker	[34]
F-SH	BS	UC	78 UC (58); 58 HC (TW & RI-EIA)	↓ in aUC vs. iUC and HC; TW -0.55	UC activity	[58]
P-SH	T-H	CD, UC	12 CD (ns/HBI≥8) 5 UC (ns/MDAI≥6); 12 HC (NM)	No associations	none	[44]
aF-SH	BP	CD	51 CD (ai/HBI, ns); 27 HC	↓ in iCD vs. HC; ↓ in ileocol. vs. col.; CRP -0.45	CD marker	[54]
alb	saliva	CD	28 CD (ns/CDAI, ns); 20 HC	↓ in CD	CD marker	[17]
alb	BP	IBD	167 IBD (132/CDAI≥150 or CAI>5; 100 CD and 67 UC); 45 HC	↓ in aIBD vs. HC; ↓ in aIBD vs. iIBD	IBD activity	[24]
alb	BS	CD, UC	68 UC (33/MDAI, ns); 50 CD (38/CDAI, ns); 45 HC	↓ in aUC and iUC vs. HC; ↓ in aCD and iCD vs. HC; ↓ in aCD vs. iCD	IBD marker CD activity	[51]
alb	BS	CD	14 CD (aa/CDAI, ns); 52 HC; IS	↓ in aCD vs. HC	aCD marker	[55]
alb	BS	IBD	19 UC (aa/RI, ns); 16 CD (aa/ CDAI≥150); 30 HC	↓ in IBD; for stratified IBD severity: 0.41	aIBD marker	[25]
alb	BS	CD, UC	35 UC (ns/RI≥5); 33 CD (ns/CDAI≥150); 65 HC	↓ in IBD, CD and UC vs. HC	IBD marker	[52]
alb	BS	CD	30 CD (ns/CDAI≥150); 66 HC	↑ in aCD	aCD marker	[50]
alb	BS	CD, UC	40 CD (ns/CDAI, ns); 40 UC (ns/RI-EAI, ns); 80 HC	↓ in CD vs. UC and HC	CD marker; $Diff_{CD/UC}$	[20]
alb	BP	CD	55 CD (35/ CDAI≥150); 25 GIS	↓ in aCD vs. GIS; ↓ in aCD vs. iCD; CDAI -0.76; CRP -0.41	$Diff_{CD/GIS}$ CD activity	[111]
alb	BS	CD	71 CD (53/ CDAI, ns); 125 HC	↓ in CD; gradually ↓ through: iCD/miCD/moCD/sCD; CDAI -0.41; CRP -0.34	CD marker CD activity	[53]

alb	BS	UC	66 UC (MDAI; mild≤5>advanced); 24 HC	n/a; ↓ in females	none	[46]
alb	BP	CD	51 CD (ai/HBI, ns); 27 HC	↓ in iCD vs. HC	CD marker	[54]
MT	BS	UC	15 UC; 15 UC+D; 15 HC	↑ in UC and UC+D vs. HC tended to be ↑ in UC+D vs. UC	UC marker	[69]
Phb2	T-I	UC	96 UC (ns/MDAI, ns); 38 HC (NM)	↓ in UC vs. HC; MDAI 0.36; HA 0.22; EA 0.28; CRP 0.28; ESR 0.22[3]	UC marker	[104]
Hpx	T-M	CD, UC	25 pediatric CD (aa/PCDAI, ns); 22 pediatric UC (aa/PUCAI, ns); 24 GIS	↑in CD in DeC (2.1x) and in TI (4.1x) and ↑in UC in AsC (2.2x)[4]	$Diff_{CD/UC}$	[106]
Trf	saliva	CD	28 CD (ns/ CDAI, ns); 20 HC	Insignificantly lower in CD	none	[17]

[1], number of patients (number of patients with active disease/scoring system≥cut-off for active disease); [2], data presented as ↑ increased or ↓decreased levels between indicated groups and as correlation coefficients preceded by the variable; [3], scatterplots do not show the correlations; [4], data showing fold change in expression and analyzed separately for three bowel fragments: AsC=ascending colon, DeS=descending colon, TI=terminal ileum;ns, not specified; aa, all active; ai, all inactive; n/a, no association; ileocol., ileocolonic; col., colonic; F-SH, free thiol groups; P-SH, protein thiols; aF-SH, free thiols adjusted to albumin; MT, metallothionein; Phb2, prohibin 2; Hpx, hemopexin; Trf, transferrin; HS, healthy controls; CD, Crohn's disease; UC, ulcerative colitis; IBD, inflammatory bowel diseases; aCD, active CD; iCD, inactive CD; miCD, mild CD; moCD, moderate CD; sCD, severe CD; aUC, active UC; iUC, inactive UC; aIBD, active IBD; iIBD, inactive IBD; D, dysplasia; NM, normal mucosa; GIS, non-IBD patients with gastrointestinal symptoms; $Diff_{CD/UC}$; differential marker for CD and UC; $Diff_{CD/GIS}$; differential marker for CD and GIS; CDAI, Crohn's disease activity index, MDAI, Mayo disease activity index; PCDAI, pediatric Crohn's disease activity index; PUCAI, pediatric ulcerative colitis index; RI, Rachmilewitz index; RI-EAI, Rachmilewitz endoscopic activity index; EA, endoscopic activity; HA, histopathological activity; HBI, Harvey-Bradshaw index; CAI, colitis activity index; TW, Truelove-Witt index; CRP, C-reactive protein; ESR, erythrocyte sedimentation rate; IS, interventional study; T-I, tissue-based marker determined with immunohistochemistry (IHC); T-M, tissue metaproteomics – aspirates collected from mucosal-luminal interface for the analysis of microbial and human proteins; BS, blood-based marker determined in serum; BP, blood-based marker determined in plasma; T-H, tissue-based marker determined in homogenates

Table S4. Low molecular weight antioxidants as potential biomarkers in inflammatory bowel diseases

**Analyte**	**Type**	**Disease**	**Evaluated population[1]**	**Findings[2]**	**Potential**	**Ref.**
Se	BP	CD	37 CD (26/ CDAI≥150); 37 HC	No association	none	[43]
Se	BP	IBD	167 IBD (132; CDAI≥150 or CAI>5; 100 CD and 67 UC); 45 HC	Tended to be lower in aIBD vs. HC (p=0.082)	none	[24]
Se	BW	CD	20 CD (ns/ CDAI, ns); 16 HC	No association	none	[83]
Se	BS	CD, UC	53 UC (aa/ns); 53 CD (36/ns); 30 HC	↓ in IBD vs. HC; ↓ in CD vs. UC; ↓ with UC severity (stratified); ↓ in left-sided UC vs. proctosigmoiditis	IBD marker $Diff_{CD/UC}$ UC severity	[82]
Zn	BW	IBD	167 IBD (132; CDAI≥150 or CAI>5; 100 CD and 67 UC; 45 HC	No association	none	[24]

Zn	BS	IBD	16 CD (aa/CDAI≥150); 19 UC (aa/ RI, ns); 30 HC	↓ in IBD	aIBD marker	[25]
Zn	BP	CD, UC	20 CD (ns); 20 UC (ns); 50 HC	No association	none	[26]
TAS	BP	CD	20 CD (aa/ CDAI>150); 134 HC	↓ in CD; ↑ normalize post-surgery; CRP -0.65; ESR -0.56	CD marker	[72]
TAS (crocin)	BS	CD, UC	97 CD (35/CDAI, ns); 94 UC (43/SCCAI, ns); 72 HC	↓ in UC and CD vs. HC; ↓ in aCD vs. iCD; ↓ in left-sided and pancolitis vs. proctitis n/a with activity, CRP or ESR	IBD marker CD activity	[70]
cTAS (crocin)	BS	CD, UC	97 CD (35/CDAI, ns); 94 UC (43/SCCAI, ns); 72 HC	↓ in UC and CD vs. HC; ↓ in left-sided and pancolitis vs. proctitis; n/a with activity, CRP or ESR	IBD marker	[70]
TAS (FRAP)	saliva	CD, UC	16 CD (ns); 16 UC (ns); 16 HC	↓ in CD vs. HC	CD marker	[16]
TAS (FRAP)	saliva	CD	28 CD (ns/CDAI, ns); 20 HC	↓ in CD	CD marker	[17]
TAS (DD)	BP	UC	20 UC (11/RI, ns); 20 HC	↓ in UC; n/a with activity; CRP -0.72; ESR -0.69	UC marker	[23]
TAS (ABTS)	BS	CD	15 pediatric CD (ai/PCDAI, ≥10); 15 pediatric HC; IS	n/a; PCDAI: -0.83; CRP -0.49; CAL -0.58	CD activity	[33]
TAS (ns)	BS	CD, UC	40 CD (ns/CDAI, ns); 40 UC (ns/RI-EAI, ns); 80 HC	↓ in CD and UC vs. HC	IBD marker	[20]
TAS (FRAP)	BP	CD	55 CD (35/ CDAI≥150); 25 GIS	↓ in aCD vs. GIS; ↓ in aCD vs. iCD; CDAI -0.57; CRP -0.46	$Diff_{CD/GIS}$ CD activity	[111]
TAS (FRAP)	saliva	CD	58 CD (36/CDAI≥150); 26 HC	n/a; CDAI -0.4; CRP -0.4	none	[37]
TAS (S_{IND}2)	T-H	CD	45 CD (aa/ns); 30 IBS; IS	↑ in CD vs. IBS	$Diff_{CD/IBS}$	[110]
TAS (FRAP)	BS	IBD	30 IBD (CDAI for CD & LI for UC); 30 HC	↓ in IBD; n/a with activity, ESR, CRP, or CAL	IBD marker	[40]
TAS (ns)	BP	IBD	71 pediatric IBD (47/PCDAI for CD and PUCAI for UC; 35 CD and 36 UC); 29 GIS	No association	none	[22]
TAS (ns)	BP	CD	25 adult CD (ns/CDAI≥150); 88 HC; 21 pediatric CD (ns/PCDAI, ns); 11 HC	n/a; PCDAI –0.53	CD activity	[35]

TAS (ABTS)	BP	CD, UC	221 CD (ns); 123 UC (ns); 294 HC	↑ in CD and UC vs. HC; ↑B3 vs. B1 CD behavior[5]	IBD marker penetrating CD	[71]
t-bil	BS	CD	30 CD (ns/CDAI≥150); 66 HC	↑ in CD vs. HC; ↑ in aCD but ↓ in iCD	CD marker CD activity	[50]
t-bil	BP	CD	55 CD (35/ CDAI≥150); 25 GIS	↓ in aCD vs. GIS; ↓ in aCD vs. iCD; CDAI -0.52; CRP -0.48	$Diff_{CD/GIS}$ CD activity	[111]
t-bil	BS	IBD	242 CD (ns/CDAI, ns); 211 UC (ns/MDAI, ns); 255 HC	↓ in IBD; ↓ in UC-E3 (extension); ↓ in colonic CD (L2); ↓ in penetrating CD(B3)[5] CDAI -0.68; CRP[3] -0.45; ESR[3] -0.45; CAL[3] -0.39; MDAI -0.43; CRP[4] -0.47; ESR[4] -0.46; CAL[4] -0.46	IBD marker UC severity penetrating CD IBD activity	[75]
t-bil	BS	CD	71 CD (53/CDAI, ns); 125 HC	↓ in CD; gradually ↓ through: iCD/miCD/moCD/sCD; CDAI -0.62; CRP -0.36	CD marker CD activity	[53]
i-bil	BS	CD	71 CD (53/CDAI, ns); 125 HC	↓ in CD; gradually ↓ through: iCD/miCD/moCD/sCD; CDAI -0.62; CRP -0.37	CD marker CD activity	[53]
d-bil	BS	CD	71 CD (53/CDAI, ns); 125 HC	↓ in CD; gradually ↓ through: iCD/miCD/moCD/sCD; CDAI -0.30; CRP -0.37	CD marker CD activity	[53]
GSH+GSSG	BP	CD, UC	20 CD (ns); 20 UC (ns); 50 HC	No association	none	[26]
GSH	BP	CD	22 CD pediatric (13/CDAI≥150); 10 HC	↑ in CD; insignificantly ↑ in aCD vs. iCD	CD marker	[86]
GSH	BP	CD	10 CD (8/CDAI, >150); 10 HC; IS	No association	none	[88]
GSH	BE	CD	25 adult CD (ns/CDAI≥150); 88 HC; 21 pediatric CD (ns/PCDAI, ns); 11 HC	↓ in complications (abscess, fistula or stenosis)	Compl.	[35]
GSH	BS	UC	15 UC; 25 UC+D; 15 HC	↓ in UC UC+D vs. HC ↓ in UC+D vs. UC	progression UC marker	[69]
GSH	BP	CD	55 CD (35/ CDAI≥150); 25 GIS	↓ in aCD vs. GIS; ↓ in aCD vs. iCD; CDAI -0.76; CRP -0.41	$Diff_{CD/GIS}$ CD activity	[111]
GSH	T-H	CD	20 CD; 18 infl. and 14 non-infl. biopsies; 16 HC (NM)	↓ in infl. vs. non-infl. and NM	MI	[83]

GSH	BE	UC	81 UC (ns/AI≥150); 85 HC	↓ in UC vs. HC; n/a with activity	UC marker	[32]
GSH	saliva	CD	58 CD (36/CDAI≥150); 26 HC	↓ aCD vs. iCD and HC; CDAI -0.5; CRP -0.6	CD activity	[37]
Cys	BP	CD	10 CD (8/CDAI>150); 10 HC; IS	↓ in CD	CD marker	[88]
UA	saliva	CD	28 CD (ns/CDAI, ns); 20 HC	↓ in CD	CD marker	[17]
UA/Cr			334 CD (174/CDAI>150 or and/or (HBI≥5) and/or CRP ≥5mg/L) 101 UC (80/MDAI≥6 and/or CRP≥5mg/L); 51 HC	↑aCD vs. iCD and HC; ↑aUC and iUC vs. HC; ↑ in CD-L2 vs. L1 and L3[5] CDAI 0.18; HBI 0.15; CRP[3] 0.53; n/a for UC	IBD marker CD activity	[78]
UA	BS	CD	71 CD (53/ CDAI, ns); 125 HC	↓ in CD; CDAI -0.30; CRP -0.34	CD marker	[53]

[1], number of patients (number of patients with active disease/scoring system≥cut-off for active disease); [2], data presented as ↑ increased or ↓decreased levels between indicated groups and as correlation coefficients preceded by the variable; [3], correlation in CD patients; [4], correlation in UC patients; [5], Montreal classification; ns, not specified; aa, all active; ai, all inactive; n/a, no association; Compl. , complications; inflam., inflamed; Se, selenium; Zn, zinc; TAS, total antioxidant status; cTAS, corrected total antioxidant status (after subtraction of the interactions due to endogenous uric acid, bilirubin and albumin); DD, o-dianizidine method; ABTS, ; $S_{IND}2$, the sum of light energy over 2 min depends on activity of the antioxidant and antiradical defense system; FRAP, assay which measures the reduction of Fe3+ (ferric ion) to Fe2+ (ferrous ion) in the presence of antioxidants; t-bil, total bilirubin; i-bil, indirect bilirubin; d-bilirubin; GSH, reduced glutathione; GSSG, oxidized glutathione; Cys, cysteine; UA, uric acid; UA/Cr, uric acid adjusted to creatinine; HS, healthy controls; CD, Crohn's disease; UC, ulcerative colitis; IBD, inflammatory bowel diseases; a IBD, active IBd; iIBD, inactive IBD; miCD, mild CD; moCD, moderate CD; sCD, severe CD; aCD, active CD; iCD, inactive CD; aUC, active UC; iUC, inactive UC; IBS, irritable bowel syndrome; D, dysplasia; NM, normal mucosa; GIS, non-IBD patients with gastrointestinal symptoms; MI, mucosal inflammation; $Diff_{CD/UC}$; differential marker for CD and UC; $Diff_{CD/GIS}$; differential marker for CD and GIS; $Diff_{CD/IBS}$; differential marker for CD and IBS; CDAI, Crohn's disease activity index, MDAI, Mayo disease activity index; PCDAI, pediatric Crohn's disease activity index; PUCAI, pediatric ulcerative colitis index; RI, Rachmilewitz index; RI-EAI, Rachmilewitz endoscopic activity index; LI, Lichtiger index; HBI, Harvey-Bradshaw index; AI, activity index defined by authors in their paper; SCCAI, Simple Clinical Colitis Activity Index; CRP, C-reactive protein; ESR, erythrocyte sedimentation rate; CAL, fecal calprotectin; IS, interventional study; BS, blood-based marker determined in serum; BE, blood –based marker determined in erythrocytes; BP, blood-based marker determined in plasma; BW, blood-based marker determined in whole blood; T-H, tissue-based marker determined in homogenates.

Table S5. Vitamins and related compounds as potential markers in inflammatory bowel diseases

Analyte	Type	Disease	Evaluated population[1]	Findings[2]	Potential	Ref.
Vit.A	BP	CD	22 pediatric CD (13/CDAI≥150); 10 HC	↓ in CD	CD marker	[86]
Vit.A	BP	UC, CD	46 UC (15/PTI>2); 37 CD (10/CDAI>150); 386 HC	↓ in UC and CD vs. HC; n/a with activity, CRP or ESR; ↓ if BMI<20	IBD marker Malnutrition	[87]
Vit.A	BP	CD	20 CD (aa/CDAI>150); 134 HC	↓ in CD; ↑ post-surgery but still ↑ than in HC	CD marker	[72]
Vit.A	BP	CD	37 CD (26/CDAI≥150); 37 HC	No associations	none	[43]
Vit.A	BP	IBD	51 IBD (ns); 67 AD (90% tubulare); 136 CRC; 79 HC	↑ in IBD vs. CRC	$Diff_{IBD/CRC}$	[89]
Vit.E	BP	CD	22 pediatric CD (13/CDAI, ≥150); 10 HC	No association	none	[86]
Vit.E	BP	UC, CD	46 UC (15/PTI>2); 37 CD (10/CDAI>150); 386 HC	↓ in UC and CD vs. HC; ↓ in aUC vs. iUC; n/a with activity, CRP, ESR; ↓if BMI<20	IBD marker UC activity Malnutrition	[87]
Vit.E	BP	CD	20 CD (aa/CDAI>150); 134 HC	↓ in CD; insign. ↑ post-surgery	CD marker	[72]
Vit.E	BP	CD	37 CD (26/CDAI≥150); 37 HC	No associations	none	[43]
Vit.E	BP	CD	10 CD (8/ CDAI>150); 10 HC	Tended to be ↓ in CD	none	[88]
Vit.E	BP	IBD	51 IBD (ns); 67 AD (90% tubulare); 136 CRC; 79 HC	No association	none	[89]

Vit.E	BP	IBD	167 IBD (132/CDAI≥150 or CAI>5; 100 CD and 67 UC); 45 HC	No associations	none	[24]
Vit.C	BP	CD	37 CD (26/ CDAI≥150); 37 HC	↓ in CD; n/a with activity	CD marker	[43]
Vit.C	BP	CD	10 CD (8/CDAI>150); 10 HC; IS	Tended to be ↓ in CD	none	[88]
Vit.C	BP	IBD	167 IBD (132/CDAI≥150 or CAI>5; 100 CD and 67 UC); 45 HC	↓ in IBD, both iIBD and aIBD	IBD marker	[24]
Vit.C	BP	IBD	51 IBD (ns); 67 AD (90% tubulare); 136 CRC; 79 HC	↑ in IBD vs. CRC	$Diff_{IBD/CRC}$	[89]
α-CA	BP	UC, CD	46 UC (15/PTI>2); 37 CD (10/CDAI>150); 386 HC	No associations	None	[87]
α-CA	BP	CD	37 CD (26/ CDAI≥150); 37 HC	↓ in CD; n/a with activity	CD marker	[43]
α-CA	BP	IBD	167 IBD (132/CDAI≥150 or CAI>5; 100 CD and 67 UC); 45 HC	↓ in IBD, both iIBD and aIBD	IBD marker	[24]
β-CA	BP	CD	22 pediatric CD (13/CDAI≥150); 10 HC	No association	none	[86]
β-CA	BP	UC, CD	46 UC (15/PTI>2); 37 CD (10/CDAI>150); 386 HC	↓ in UC and CD vs. HC; ↓ in aCD vs. iCD; n/a with activity, CRP, ESR	IBD marker CD activity	[87]
β-CA	BP	CD	37 CD (26/ CDAI≥150); 37 HC	↓ in CD; n/a with activity	CD marker	[43]
β-CA	BP	CD	10 CD (8/CDAI>150); 10 HC; IS	↓ in CD	CD marker	[88]
β-CA	BP	IBD	167 IBD (132/CDAI≥150 or CAI>5; 100 CD and 67 UC); 45 HC	↓ in IBD, both iIBD and aIBD	IBD marker	[24]
β-CA	BS_L	CD	43 CD (16/CDAI, >150); 15 HC	↓ CD vs. HC; ↓ aCD vs. iCD	CD marker CD activity	[41]
ΣCA	BP	UC, CD	46 UC (15/PTI>2); 37 CD (10/CDAI>150); 386 HC	↓ in UC and CD vs. HC; ↓ in aUC vs. iUC; ↓ in aCD vs. iCD; CDAI -0.36; PTI -0.33; n/a with CRP, ESR; ↓if BMI<20	IBD marker UC activity CD activity Malnutrition	[87]
ΣCA	BP	IBD	167 IBD (132/CDAI≥150 or CAI>5; 100 CD and 67 UC); 45 HC	↓ in IBD, both iIBD and aIBD	IBD marker	[24]
Lut	BP	UC, CD	46 UC (15/PTI>2); 37 CD (10/CDAI>150); 386 HC	↓ in aCD vs. iCD; n/a with activity, CRP or ESR	CD activity	[87]
Lut	BP	CD	37 CD (26/ CDAI≥150); 37 HC	No associations	none	[43]
Zea	BP	UC, CD	46 UC (15/PTI>2); 37 CD (10/CDAI>150); 386 HC	↓ in aUC vs. iUC; ↓ in aCD vs. iCD; n/a with activity, CRP, ESR	UC activity CD activity	[87]
Zea	BP	CD	37 CD (26/ CDAI≥150); 37 HC	No associations	none	[43]
Lyc/Zea	BP	IBD	167 IBD (132/CDAI≥150 or CAI>5; 100 CD and 67 UC); 45 HC	↓ in IBD, both iIBD and aIBD; ↓ in aIBD vs. iIBD (p=0.052)	IBD marker	[24]
Lyc	BP	UC, CD	46 UC (15/PTI>2); 37 CD (10/CDAI>150); 386 HC	↓ in UC and CD vs. HC; ↓ in aUC vs. iUC; ↓ in aCD vs. iCD; n/a with activity, CRP, ESR	IBD marker UC activity CD activity	[87]
Lyc	BP	CD	37 CD (26/ CDAI≥150); 37 HC	↓ in CD; n/a with activity	CD marker	[43]
Lyc	BP	IBD	167 IBD (132/CDAI≥150 or CAI>5; 100 CD and 67 UC); 45 HC	↓ in IBD, both iIBD and aIBD; ↓ in aIBD vs. iIBD	IBD marker IBD activity	[24]
β-CX	BP	UC, CD	46 UC (15/PTI>2); 37 CD (10/CDAI>150); 386 HC	↓ in UC and CD vs. HC; ↓ in aUC vs. iUC; n/a with activity, CRP or ESR	IBD marker UC activity	[87]
β-CX	BP	CD	37 CD (26/ CDAI≥150); 37 HC	↓ in CD; n/a with activity	CD marker	[43]
β-CX	BP	IBD	167 IBD (132/CDAI≥150 or CAI>5; 100 CD and 67 UC); 45 HC	↓ in IBD, both iIBD and aIBD	IBD marker	[24]

[1], number of patients (number of patients with active disease/scoring system≥cut-off for active disease); [2], data presented as ↑ increased or ↓decreased levels between indicated groups and as correlation coefficients preceded by the variable; ns, not specified; aa, all active; n/a, no association; insign., non-significant; Vit, vitamin; β-CA, β-carotene; α-CA, α-carotene; Σ-CA, total carotenoids; Lut, lutein; Lyc, lycopene; Zea, zeaxanthin; β-CX, β-cryptoxanthin; HS, healthy controls; CD, Crohn's disease; UC, ulcerative colitis; IBD, inflammatory bowel disease; aIBD; active IBD; iIBD, inactive IBD; aCD, active CD; iCD, inactive CD; aUC, active UC; iUC, inactive UC; CRC, colorectal cancer; AD, adenoma; BMI, body mass index; $Diff_{IBD/CRC}$; differential marker for IBD and CRC; CDAI, Crohn's disease activity index; PTI, Powell-Tuck index; CAI; colitis activity index; PCDAI, pediatric Crohn's disease activity index; PUCAI, pediatric ulcerative colitis index; CRP, C-reactive protein; ESR, erythrocyte sedimentation rate; IS, interventional study; BS, blood-based marker determined in serum; BP, blood-based marker determined in plasma; BL, blood-based marker determined in leukocytes; BS_L, blood-based marker determined in lipid fraction of serum

Table S6. Lipid peroxidation markers as potential biomarkers in inflammatory bowel disease

Analyte	Type	Disease	Evaluated population[1]	Findings[2]	Potential	Ref.
MDA (TBARS)	BP	CD	22 pediatric CD (13/CDAI≥150); 10 HC	↑ in CD; insign. ↑ in aCD vs. iCD	CD marker	[86]
MDA (TBARS)	BP	CD	20 CD (aa/CDAI>150); 134 HC	↑ in CD; ↓ post-surgery but ↑ vs. HC; CRP 0.6; ESR 0.51	CD marker	[72]
MDA (TBARS)	BS	CD, UC	5 CD (1/HBI, ns); 7 UC (low activity/SCCAI, ns); 12 HC	↑ in IBD vs HC; ↑ in CD and UC vs. HC	IBD marker	[93]
MDA (TBARS)	BS	UC	30 UC (aa/TW, ns); 30 HC	↑ in UC; n/a with severity, CRP, ESR	UC marker	[17,]
MDA (TBARS)	BP	CD	25 adult CD (ns/CDAI≥150); 88 HC; 21 pediatric CD (ns/PCDAI, ns); 11 HC	↑ in complications (abscess, fistula or stenosis)	Compl. CD marker	[35]
MDA (TBARS)	saliva	CD, UC	16 CD (ns); 16 UC (ns); 16 HC	↑ in CD vs. HC	CD marker	[16]
MDA (TBARS)	saliva	CD	28 CD (ns/ CDAI, ns); 20 HC	↑ in CD	CD marker	[17]
MDA (TBARS)	saliva	CD	58 CD (36/CDAI≥150); 26 HC	↑ aCD vs. iCD and HC; CDAI 0.8; CRP 0.7	aCD marker CD activity	[37]
MDA[3] (TBARS)	BE	IBD	91 IBD (43/CDAI & TW); 45 HC	↑ in aIBD and iIBD vs. HC; n/a with activity	IBD marker	[34]
MDA (TBARS)	BP	UC	59 UC (33/RI≥4); 51 HC	No associations	none	[92]
MDA (TBARS)	BS	IBD	14 CD (8/CDAI≥150); 27 UC (13/MTS≥4); 18 HC	↑ in aIBD vs. HC	IBD activity	[38]
MDA (TBARS)	T-H	CD, UC	12 CD (ns/HBI≥8) 5 UC (ns/MDAI≥6); 12 HC (NM)	↑ in CD and UC vs. HC	IBD marker	[44]
MDA (TBARS)	BS	CD	27 CD (8/HBI≥5); 22 HC	↑ in CD	CD marker	[94]
MDA (TBARS)	BP	CD, UC	221 CD (ns); 123 UC (ns); 294 HC	No associations	none	[71]
MDA (TBARS)	BS	IBD	30 IBD (CDAI for CD & LI for UC); 30 HC	↑ in IBD; n/a with activity, CRP, ESR or CAL	IBD marker	[40]
MDA (TBARS)	BP	CD, UC	20 CD (ns); 20 UC (ns); 50 HC	n/a; CRP[5] -0.63	none	[26]

MDA (TBARS)	BP	CD, UC	35 UC (13/RI, ns); 12 CD (6/HBI, ns); 30 HC	No association	none	[39]
MDA (TBARS)	BP	CD	43 CD (16/CDAI>150); 15 HC	↑ CD vs. HC; ↑ in aCD vs. iCD and HC	CD marker CD activity	[41]
MDA (TBARS)	BP	CD	25 aCD (HBI≥5)→19 rCD[4]; 20 iCD; HC	↑ in aCD, iCD, and rCD, vs. HC; ↑ in aCD vs. iCD	CD marker; CD activity	[36]
HNE	BS	CD, UC	5 CD (1/HBI, ns); 7 UC (low activity/SCCAI, ns); 12 HC	↑ in IBD vs HC; ↑ in CD and UC vs. HC	IBD marker	[93]
DIEN	BS	CD	14 CD (aa/ CDAI, ns); 52 HC	Not associations	none	[55]
DIEN	T-H	CD, UC	12 CD (ns/HBI≥8); 5 UC (ns/MDAI≥6); 12 HC (NM)	↑ in CD and UC vs. HC	IBD marker	[44]
maxPR	BP	CD	20 CD (aa/CDAI>150); 134 HC	No associations	none	[72]
LPO	BS	CD	15 pediatric CD (aa/PCDAI≥10); 15 HC; IS	No associations	none	[33]
LPO	BE	UC	81 UC (ns/AI≥150); 85 HC	↑ in UC vs. HC; n/a activity	UC marker	[32]
LPO	BS	CD	43 CD (16/CDAI>150); 15 HC	↑ CD vs. HC; ↑ in aCD vs. iCD and HC	CD marker CD activity	[41]
LOOH	T-H	CD	45 CD (aa/ns); 30 IBS; IS	↑ in CD vs. IBS	$Diff_{CD/IBS}$	[110]
LOOH	BP	CD, UC	20 CD (ns); 20 UC (ns); 50 HC	↓ in CD and UC vs. HC	none	[26]
LOOH	BP	CD	52 CD (37/CDAI≥150); 99 HC	No associations	none	[21]
pentane	EA	CD	37 CD (26/CDAI≥150); 37 HS	↑ in CD; n/a with activity	CD marker	[43]
ethane	EA	CD	37 CD (26/CDAI≥150); 37 HC	↑ in CD; n/a with activity	CD marker	[43]
8-iso-PGF2a	urine	CD	23 CD (12/CDAI≥150); 23 HC	↑ in CD; tended to be ↑ in aCD vs. iCD (p=0.09); CRP 0.45	CD marker	[95]
8-iso-PGF2a	BP	CD	37 CD (26/CDAI≥150); 37 HC	↑ in CD; n/a with activity	CD marker	[43]
8-iso-PGF2a	urine	CD	15 pediatric CD (aa/PCDAI≥10); 15 HC; IS	No associations	none	[33]
8-iso-PGF2a	BS	CD, UC	31 CD (ns/CDAI≥150) 32 UC (ns/DAI≥6); 64 HC	↑ in aUC and iUC vs. HC ↑ in aUC vs. iUC ↑ in aCD and iCD vs. HC ↑ in CD than UC; DAI 0.40	IBD marker UC activity $Diff_{CD/UC}$	[96]
8-iso-PGF2a	urine	CD, UC	57 CD (28/CDAI≥150); 67 UC (19/RI≥4); 37 HC; IS	↑ in UC and CD vs. HC; ↑ in aIBD vs. iIBD	IBD marker IBD activity	[97]
oxLDL	BP	CD	52 CD (37/CDAI≥150); 99 HC	↓ in aCD vs. HC	aCD marker	[21]

oxLDL	BP	IBD	71 pediatric IBD (47/PCDAI for CD and PUCAI for UC; 35 CD and 36 UC); 29 GIS	↑ in iIBD vs. aIBD; ↑ with duration and cholesterol	IBD activity	[22]
OLAB	BP	CD	52 CD (37/CDAI≥150); 99 HC	n/a; CDAI 0.31	none	[21]
OLAB	BP	IBD	71 pediatric IBD (47/PCDAI for CD and PUCAI for UC; 35 CD and 36 UC); 29 GIS	No association	none	[22]

[1], number of patients (number of patients with active disease/scoring system≥cut-off for active disease); [2], data presented as ↑ increased or ↓decreased levels between indicated groups and as correlation coefficients preceded by the variable; [3], determined following HPLC separation; [4], patients with active disease were followed until they have achieved remission; [5] correlation in CD patients; ns, not specified; aa, all active; n/a, no association; insign., non-significantly; MDA (TBARS), malonodialdehyde determined as thiobarbituric acid-reactive substances (TBARS); HNE, 4-hydroxy-2-nonenal; DIEN, conjugated diens; maxPR, the maximal rate of oxidation (depends on compounds available for peroxidation ; LPO, lipid peroxides; LOOH, lipid hydroperoxides; 8-iso-PGF2a, 8-iso-prostaglandin F2 alpha; oxLDL, oxidized light-density lipoprotein; OLAB, antibodies directed against oxLDL; HS, healthy controls; CD, Crohn's disease; UC, ulcerative colitis; IBD, inflammatory bowel diseases; iIBD, inactive IBD; aIBD, active IBD; aCD, active CD; iCD, inactive CD; rCD, remission CD; aUC, active UC; iUC, inactive UC; NM, normal mucosa; GIS, non-IBD patients with gastrointestinal symptoms; Diff$_{CD/UC}$; differential marker for CD and UC; Diff$_{CD/IBS}$; differential marker for CD and IBS; CDAI, Crohn's disease activity index, MDAI, Mayo disease activity index; PCDAI, pediatric Crohn's disease activity index; PUCAI, pediatric ulcerative colitis index; RI, Rachmilewitz index; LI, Lichtiger index; HBI, Harvey-Bradshaw index; AI, activity index defined by authors in their paper; TW, Truelove-Witt index; MTS, Mayo total score; SCCAI, Simple Clinical Colitis Activity Index; CRP, C-reactive protein; ESR, erythrocyte sedimentation rate; CAL, fecal calprotectin; IS, interventional study; BS, blood-based marker determined in serum; BE, blood –based marker determined in erythrocytes; BP, blood-based marker determined in plasma; T-H, tissue-based marker determined in homogenates; EA, exhaled air.

Table S7. Markers of oxidative damage to proteins as potential biomarkers in inflammatory bowel diseases

**Analyte**	**Type**	**Disease**	**Evaluated population[1]**	**Findings[2]**	**Potential**	**Ref.**
PC	T-H	CD, UC	22 UC (15/ ns), paired biopsies inf. & non-infl. colon (n=6 aUC); 11 CD (6/ns); 14 specific colitis (infl.); 10 GIS	↑ in aUC and iUC vs. GIS; ↑ in aCD and iCD vs. GIS; ↑ in spec. colitis vs. GIS; ↑ in aUC/aCD vs. iUC/iCD; ↑ in infl. and non-infl. aUC vs. iUC and GIS; ↑ in infl. aUC vs. non-infl.; If stratified by severity into: CON/iIBD/miIBD/moIBD/sIBD, r=0.81	IBD marker IBD activity Diff$_{IBD/GIS}$ MI in UC	[19]
PC	T-H	UC	12 UC: 6 non-progressors (UC) and 6 progressors (UC+LGD, HGD or UCAC)	↑ in UC+LGD and UC+HGD vs. UC; tended to be ↑ also in non-dysplastic tissue from progressors (p=0.08)	Progression	[123]
PC	BP	CD, UC	221 CD (ns); 123 UC (ns); 294 HC	No association	none	[71]
nTyr	T-H	CD, UC	22 UC (15/ ns), paired biopsies inf. & non-infl. colon (n=6 aUC); 11 CD (6/ns); 14 specific colitis (infl.); 10 GIS	↑ in aUC and iUC vs. GIS; ↑ in aCD and iCD vs. GIS; ↑ in spec. colitis vs. GIS; ↑ in aUC/aCD vs. iUC/iCD; ↑ in infl. and non-infl. aUC vs. iUC and GIS; ↑ in infl. aUC vs. non-infl.; If stratified by severity into: CON/iIBD/miIBD/moIBD/sIBD, r=0.84	IBD marker IBD activity Diff$_{IBD/GIS}$ MI in UC	[19]
nTyr	T-H	CD, UC	18 UC biopsies and 22 CD biopsies	No difference between CD and UC	none	[100]
nTyr	BS	CD, UC	57 UC (38/ns); 62 CD (42/ns); 20 HC	↑ in aUC vs. iUC ↓ in iUC vs. CON	UC marker UC activity	[100]
Cl-Tyr	T-H	CD, UC	18 UC and 22 CD biopsies	No difference between CD and UC	none	[100]

Cl-Tyr	BS	CD, UC	57 UC (38/ns); 62 CD (42/ns); 20 HC	↑ in aUC and aCD vs. HC; ↑ in aUC vs. iUC and in aCD vs. iCD	IBD marker IBD activity	[100]
AOPP	BS	UC	30 UC (aa/TW, ns); 30 HC	↑ in UC; n/a with severity, CRP, ESR	UC marker	[18]
AOPP	BS	UC	15 UC; 15 UC+D; 15 HC	↑ in UC+D and UC vs. HC ↑ in UC+D vs. UC	UC marker Progression	[69]
AOPP	BP	UC	59 UC (33/RI≥4); 51 HC	↑ in aUC vs. iUC and HC; EA 0.61	UC activity MI	[92]
AOPP	BS	CD	15 pediatric CD (ai/PCDAI, ≥10); 15 HC; IS	↑ in CD	CD marker	[33]
IMA	BS	CD, UC	39 CD (ns); 41 UC (ns), 33 HC	↑ in IBD vs. HC; ↑ in UC vs. CD and HC	IBD marker $Diff_{CD/UC}$	[101][3]

[1], number of patients (number of patients with active disease/scoring system≥cut-off for active disease); [2], data presented as ↑ increased or ↓decreased levels between indicated groups and as correlation coefficients preceded by the variable; [3], based on abstract; ns, not specified; aa, all active; ai, all inactive; n/a, no association; inflam., inflamed; spec. specific colitis; PC, protein carbonyls; nTyr, 3-nitrotyrosine; Cl-Tyr, 3-chlorotyrosine; AOPP, advanced oxidation protein products; IMA, ischemia-modified protein; HS, healthy controls; CD, Crohn's disease; UC, ulcerative colitis; IBD, inflammatory bowel disease; iIBD, inactive IBD; miIBD, mild IBD; moIBD, moderate IBD; sIBD, severe IBD; aIBD, active IBD; aCD, active CD; iCD, inactive CD; aUC, active UC; iUC, inactive UC; LGD, low grade dysplasia; HGD, high grade dysplasia; UCAC, UC-associated cancer, GIS, non-IBD patients with gastrointestinal symptoms; MI, mucosal inflammation; $Diff_{CD/UC}$; differential marker for CD and UC; $Diff_{IBD/GIS}$; differential marker for IBD and GIS;CDAI, Crohn's disease activity index, PCDAI, pediatric Crohn's disease activity index; RI, Rachmilewitz index; TW, Truelove-Witt index; CRP, C-reactive protein; ESR, erythrocyte sedimentation rate; IS, interventional study; BS, blood-based marker determined in serum; BP, blood-based marker determined in plasma; T-H, tissue-based marker determined in homogenates.

Table S8. Markers of oxidative damage to DNA as potential biomarkers in inflammatory bowel diseases

Analyte	Type	Disease	Evaluated population[1]	Findings[2]	Potential	Ref.
8-OHdG	BL	UC, CD	46 UC (15/PTI>2); 37 CD (10/CDAI>150); 386 HC	↑ in CD and UC vs. HC; n/a with activity, CRP or ESR; ↓ in ST or IS-treated patients vs. 5-ASA alone	IBD marker	[87]
8-OHdG	BL	CD	25 aCD (HBI≥5)→19 rCD[3]; 20 iCD; HC	↑ in aCD, iCD, and rCD vs. HC	CD marker	[36]
8-OHdG	T-I	UC	16 aUC; 14 UC+N (9 D, 14 UCAC); 17 NM	↑ in aUC and UC+N vs. NM	Inflam./N	[30]
8-OHdG	BL	IBD	51 IBD (ns); 67 AD (90% tubulare); 136 CRC; 79 HC	↑ in IBD vs. AD and CRC and HC	IBD marker $Diff_{IBD/N}$	[89]
εdA	T-H	UC, CD	5 CD (ns); 5 UC (ns); NM	↓ in UC vs. NM; Tended to be ↑ in CD vs. NM	UC presence	[103]
εdC	T-H	UC, CD	5 CD (ns); 5 UC (ns); NM	↑ in UC vs. NM; ↑ in CD vs. NM	IBD marker	[103]
DNA ssb (comet)	BL	UC	20 UC (11/RI, ns); 20 HC	↑ in UC; n/a with activity; CRP 0.54; ESR 0.73	UC marker	[23]
DNA ssb (comet)	BLy	CD	21 pediatric CD (ns/PCDAI, ns); 11 HC	No association	none	[35]
DNA ssb (comet)	PBMC	CD, UC	221 CD (ns); 123 UC (ns); 294 HC	↑ in CD and UC vs. HC; ↑ in UC vs. CD	IBD marker $Diff_{CD/UC}$	[71]

[1], number of patients (number of patients with active disease/scoring system≥cut-off for active disease); [2], data presented as ↑ increased or ↓decreased levels between indicated groups and as correlation coefficients preceded by the variable; [3], patients with active disease were followed until they have achieved remission; ns, not specified; n/a, no association; inflam., inflamed; 8-OHdG, 8-oxo-2'-deoxyguanosine; εdA, 1,N6-ethenodeoxyadenosine (HNE-derived etheno-DNA adduct); εdC, 3,N4-ethenodeoxycytidine (HNE-derived etheno-DNA adduct); DNA ssb, single strands breaks in DNA determined with comet assay; HS, healthy controls; CD, Crohn's disease; UC, ulcerative colitis; IBD, inflammatory bowel diseases; iIBD, inactive IBD; aIBD, active IBD; aCD, active CD; iCD, inactive CD; rCD, remission CD; aUC, active UC; iUC, inactive UC; N, neoplasms; D, dysplasia; NM, normal mucosa; UCAC, UC-associated cancer; AD, adenoma; CRC, colorectal cancer; GIS, non-IBD patients with gastrointestinal symptoms; MI, mucosal inflammation; $Diff_{CD/UC}$; differential marker for CD and UC; $Diff_{IBD/N}$; differential marker for IBD and neoplastic diseases (CRC and adenomas); ST, steroids; IS, immunosuppressants; 5'-ASA, 5-aminosalicylic acid; CDAI, Crohn's disease activity index, PCDAI, pediatric Crohn's disease activity index; RI, Rachmilewitz index; HBI, Harvey-Bradshaw index; PTI, Powell-Tuck index; CRP, C-reactive protein; ESR, erythrocyte sedimentation rate; BL, blood-based marker determined in leukocytes; BLy, blood-based

marker determined in lymphocytes; T-H, tissue-based marker determined in homogenates; T-I, tissue-based marker determined with immunohistochemistry (IHC); PBMC, peripheral blood mononuclear cells.
